# *ITGA3* Is Associated With Immune Cell Infiltration and Serves as a Favorable Prognostic Biomarker for Breast Cancer

**DOI:** 10.3389/fonc.2021.658547

**Published:** 2021-05-20

**Authors:** Yue Li, Fan Li, Xiaoyu Bai, Yanlei Li, Chunsheng Ni, Xiulan Zhao, Danfang Zhang

**Affiliations:** ^1^ Department of Pathology, Tianjin Medical University, Tianjin, China; ^2^ Department of Pathology, General Hospital of Tianjin Medical University, Tianjin, China

**Keywords:** *ITGA3*, breast cancer, methylation, extracellular matrix, tumor infiltrating lymphocyte, prognosis, diagnosis

## Abstract

**Background:**

*ITGA3* is a member of the integrin family, a cell surface adhesion molecule that can interact with extracellular matrix (ECM) proteins. The purpose of this study was to explore the significance of *ITGA3* expression in the prognosis and clinical diagnosis of breast cancer patients.

**Methods:**

Oncomine, the Human Protein Atlas (HPA) and UALCAN were used to analyze the expression of *ITGA3* in various cancers. PrognoScan, GEPIA, Kaplan–Meier plotter and Easysurv were utilized to analyze the prognosis of *ITGA3* in certain cancers. Based on TCGA data, a receiver operating characteristic (ROC) curve was used to evaluate the diagnostic performance of *ITGA3* expression. cBio-Portal and MethSurv were used to evaluate the genomic mechanism. LinkedOmics, NetworkAnalyst and Metascape were used to build the signaling network. TIMER is a web server for comprehensive analysis of tumor infiltrating immune cells and tumor infiltrating lymphocytes (TILs).

**Results:**

The expression of *ITGA3* in normal breast tissues was greater than that in breast cancer tissues at both the mRNA and protein levels. High expression of *ITGA3* was associated with better prognosis of breast cancer patients. ROC analysis indicated that *ITGA3* had significant diagnostic value. Genomic analysis revealed that promoter methylation of *ITGA3* leads to transcriptional silencing, which may be one of the mechanisms underlying *ITGA3* downregulation in BRCA. Immune infiltration analysis showed that *ITGA3* may be involved in the recruitment of immune cells.

**Conclusions:**

This study identified *ITGA3* as a novel biomarker to estimate the diagnosis and prognosis of breast cancer. In addition, *ITGA3* is involved in ECM regulation and immune cell infiltration.

## Introduction

Breast cancer is the primary killer of women. Despite long-term investigation and research, the incidence of breast cancer is still rising. Worldwide, breast cancer remains the leading cancer-related cause of disease for women (1, 2). Metastasis is considered to be the main cause of the high mortality of breast cancer (3). Breast cancer is highly heterogeneous (4), mainly in terms of treatment with surgery and chemotherapy. Recently, the combination of targeted therapy and immunotherapy has achieved certain results, and early data have revealed the clinical activity of programmed cell death-1/programmed death ligand-1 (*PD-1/PD-L1*) antagonists in small numbers of patients with metastatic breast cancer (5). However, not all patients benefit. Therefore, creating an effective immunotherapy for all patients and looking for immunotherapy target markers is the primary task of clinical development.

*ITGA3* (integrin subunit α3), also known as integrin α3, is a member of the integrin family. Integrin is a transmembrane heterodimer composed of α and β subunits that are noncovalently bound. *ITGA3* encodes the α3 subunit, which undergoes posttranslational cleavage in the extracellular domain to produce light and heavy chains to combine with the β1 subunit, forming the integrin α3β1 that interacts with many ECM proteins, mediating cell-cell adhesion and cell-matrix adhesion, and connecting the external and internal structures of cells (6). *ITGA3* is widely expressed in normal organisms, but under the effects of oncogene induction, chromatin structure changes, high expression of growth factor and its receptor, ECM changes and other factors such as the enhanced transcription of integrins cause disordered expression that induces cancer. Studies have shown that *ITGA3* can be used as a poor prognostic factor for pancreatic cancer (7), head and neck cancer (8) and tongue squamous cell carcinoma (9). However, the expression and prognosis of *ITGA3* in breast cancer have not been reported.

In this study, we used a variety of databases to explore the expression of *ITGA3* in BRCA and its impact on prognosis, analyzed its diagnostic value and genomic and interacting mechanisms, and finally analyzed its impact on TILs. Our research provides new directions and insights into the mechanism of *ITGA3* in breast cancer and determined that *ITGA3* may be a potential prognostic-related biomarker in BRCA, offering new ideas for clinical diagnosis and application.

## Methods

### Oncomine

Oncomine (www.oncomine.org) is a cancer microarray database and web-based data-mining platform aimed at facilitating discovery from genome-wide expression analyses. Differential expression analyses comparing most major types of cancer with respective normal tissues as well as a variety of cancer subtypes and clinical-based and pathology-based analyses are available for exploration (10). In this study, we set the *P* value to 0.001, the fold change to 1.5, and all gene rankings as significance thresholds to evaluate the expression of *ITGA3* mRNA in pan-cancer.

### TIMER

TIMER (cistrome.shinyapps.io/timer) can be used to comprehensively investigate the molecular characterization of tumor-immune interactions. Levels of six tumor-infiltrating immune subsets were precalculated for 10,897 tumors from 32 cancer types. TIMER provides six major analytic modules that allow users to interactively explore the associations between immune infiltrates and a wide spectrum of factors, including gene expression, clinical outcomes, somatic mutations, and somatic copy number alterations (11). In this study, “Gene Module” was used to visualize the correlation of *ITGA3* mRNA levels with the immune cell infiltration levels in BRCA. The “Survival Module” was used to evaluate the correlation between the infiltration of immune cells and BRCA. The “SCNA Module” provides the comparison of tumor infiltration levels among tumors with different somatic copy number alterations for *ITGA3*. The “DiffExp module” was used to study the differential expression of *ITGA3* between tumor and adjacent normal tissues across all TCGA tumors.

### The Human Protein Atlas

The Human Protein Atlas (HPA) (www.proteinatlas.org) aims to map all human proteins in cells, tissues and organs. It presents a map of the human tissue proteome based on an integrated omics approach that involves quantitative transcriptomics at the tissue and organ level, combined with tissue microarray-based immunohistochemistry, to achieve spatial localization of proteins down to the single-cell level (12). In this study, we used the “Tissue Atlas”, which shows the distribution of *ITGA3* across breast tissues in the human body. The “Pathology Atlas” shows the impact of *ITGA3* protein levels on the survival of patients with breast cancer. In addition, we generated an immunohistochemical map of *ITGA3* in breast tissue and breast cancer tissue.

### UALCAN

Ualcan (ualcan.path.uab.edu/index.html) is a comprehensive web portal to perform in-depth analyses of TCGA gene expression data. UALCAN uses TCGA level 3 RNA-seq and clinical data from 31 cancer types to estimate the effects of gene expression levels and clinicopathologic features on patient survival (13). In this study, *ITGA3* expression data were obtained using the “TCGA Analysis” module of UALCAN and the “BRCA” dataset. Student’s t test was used to generate a *P* value. The *P* value cutoff was 0.05.

### PrognoScan

PrognoScan (dna00.bio.kyutech.ac.jp/PrognoScan/index.html) provides a powerful platform for evaluating potential tumor markers and therapeutic targets. It is a large collection of publicly available cancer microarray datasets with clinical annotation, as well as a tool for assessing the biological relationship between gene expression and prognosis (14). In this study, which showed the prognostic level of *ITGA3* in a variety of cancers, the Cox *P*-value cutoff was 0.05.

### GEPIA 2

GEPIA 2 (gepia2.cancer-pku.cn/#index), Gene Expression Profiling Interactive Analysis, is a web-based tool to deliver fast and customizable functionalities based on TCGA and GTEx data. GEPIA provides key interactive and customizable functions, including differential expression analysis, profiling plotting, correlation analysis, patient survival analysis, similar gene detection and dimensionality reduction analysis (15). In this study, we generated a survival map of *ITGA3* in the “survival analysis” module, and the significance level was 0.05.

### Kaplan–Meier Plotter

Kaplan–Meier plotter (kmplot.com/analysis/index.php?p=background) is a meta-analysis-based platform for the discovery and validation of survival biomarkers, including 54k genes (mRNA, miRNA, protein) related to on survival in breast, ovarian, lung, and gastric cancer (16). To analyze the prognostic value of a particular gene, the cohorts were divided into two groups according to the median (or upper/lower quartile) expression of the gene. The two groups can be compared in terms of relapse-free survival, overall survival, and distant metastasis-free survival. In this study, we analyzed the prognosis of *ITGA3* in these four cancers. The hazard ratios (HRs) with 95% confidence intervals and log-rank *P*-values were also computed.

### Easysurv

Easysurv (easysurv.net) is a web-based tool that can perform advanced survival analyses using user-derived data or data from The Cancer Genome Atlas (TCGA), which can conduct univariate analyses and grouped variable selections using multiomics data from TCGA and advanced statistical techniques suitable for high-dimensional data, including genetic data and integrated survival analysis. Through univariate analyses, ESurv can identify the prognostic significance for single genes using the survival curve (median or optimal cutoff), area under the curve (AUC) with C statistics, and receiver operating characteristics (ROC) (17). In this study, we used the univariate analysis and selected the median cutoff to generate a Kaplan–Meier plot of *ITGA3*. The *P* value cutoff was 0.05.

### ROC Curve

The diagnostic role of *ITGA3* in BRCA was assessed by receptor operating characteristic (ROC) curve analysis based on TCGA data, which were downloaded from the UCSC Xena database (xena.ucsc.edu/). In this study, we selected the GDC TCGA breast cancer cohort and extracted the gene expression RNAseq (HTSeq-Counts) data of ENSG00000005884.16 (n = 1217). A *P*-value <0.05 was considered statistically significant.

### cBio-Portal

The cBio Cancer Genomics Portal (www.cbioportal.org/) is an open-access resource for interactive exploration of multidimensional cancer genomics data sets, currently providing access to data from more than 5,000 tumor samples from 20 cancer studies (18). Copy number variation (CNV) and methylation analysis of *ITGA3* in BRCA were performed in this study.

### MethSurv

MethSurv (biit.cs.ut.ee/methsurv/) is a web tool for survival analysis based on CpG methylation patterns. MethSurv enables survival analysis for a CpG located in or around the proximity of a query gene. For further mining, cluster analysis for a query gene to associate methylation patterns with clinical characteristics and browsing of top biomarkers for each cancer type are provided (19). In this study, we verified the *ITGA3* methylation level and the methylation level under different clinical stages through a promoter probe.

### NetworkAnalyst

NetworkAnalyst (www.networkanalyst.ca/) addresses the key need to interpret gene expression data within the context of protein–protein interaction (PPI) networks (20). It can create cell-type or tissue-specific PPI networks, gene regulatory networks, gene coexpression networks and networks for toxicogenomics and pharmacogenomics studies. In this study, we used methylated genes from the cBio-Portal database to build a signaling network.

### LinkedOmics

LinkedOmics (www.linkedomics.org/login.php) contains multiomics data and clinical data for 32 cancer types and a total of 11,158 patients from The Cancer Genome Atlas (TCGA) project. It is also the first multiomics database that integrates mass spectrometry (MS)-based global proteomics data generated by the Clinical Proteomic Tumor Analysis Consortium (CPTAC) on selected TCGA tumor samples (21). In this study, we analyzed the coexpressed genes of *ITGA3* in BRCA and produced volcano maps and related heat maps.

### Metascape

Metascape (metascape.org/gp/index.html#/main/step1) is a web-based portal designed to provide a comprehensive gene list annotation and analysis resource for experimental biologists. Metascape combines functional enrichment, interactome analysis, gene annotation, and membership search to leverage over 40 independent knowledge bases within one integrated portal. Additionally, it facilitates comparative analyses of datasets across multiple independent and orthogonal experiments (22). In this study, we used the coexpressed genes of *ITGA3* from the LinkedOmics database for Gene Ontology (GO) and Kyoto Encyclopedia of Genes and Genomes (KEGG) pathway enrichment.

## Results

### *ITGA3* Expression Profiles in Pan-Carcinoma

**Figure 1:**
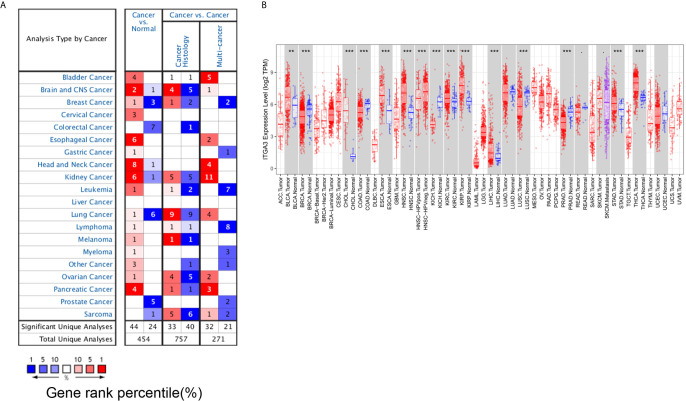
*ITGA3* transcription level in different types of tumor tissues and normal tissues. **(A)** The mRNA level of *ITGA3* in different types of tumor tissues and normal tissues in the Oncomine database (P value is 0.001, fold change is 1.5, and gene ranking of all.). **(B)** The mRNA level of *ITGA3* in different types of tumor tissues and normal tissues in TIMER database (P-value Significant Codes: 0 ≤ *** < 0.001 ≤ ** < 0.01 ≤ · < 0.1).

We primarily analyzed the transcription levels of *ITGA3* in multiple tumors and normal tissues based on the Oncomine database. We found that the mRNA expression of *ITGA3* in bladder cancer, brain and CNS cancer, cervical cancer, esophageal cancer, gastric cancer, head and neck cancer, kidney cancer, leukemia, lymphoma, melanoma, myeloma, ovarian cancer, pancreatic cancer and other cancers was higher than that in adjacent normal tissues, while in breast cancer, colorectal cancer, lung cancer, prostate cancer and sarcoma, the expression was lower than that in normal controls (**Figure 1A**).

We further used the TIMER database to evaluate which cancers have differential expression at the mRNA level. The results showed that *ITGA3* was markedly overexpressed in 13 types of cancer (BLCA, ESCA, CHOL, HNSC, KIRC, KIRP, LIHC, STAD, THCA) compared with the corresponding normal controls. In contrast, *ITGA3* was expressed at lower levels in BRCA, COAD, KICH, LUSC and PRAD than in normal controls (**Figure 1B**).

**Figure 2:**
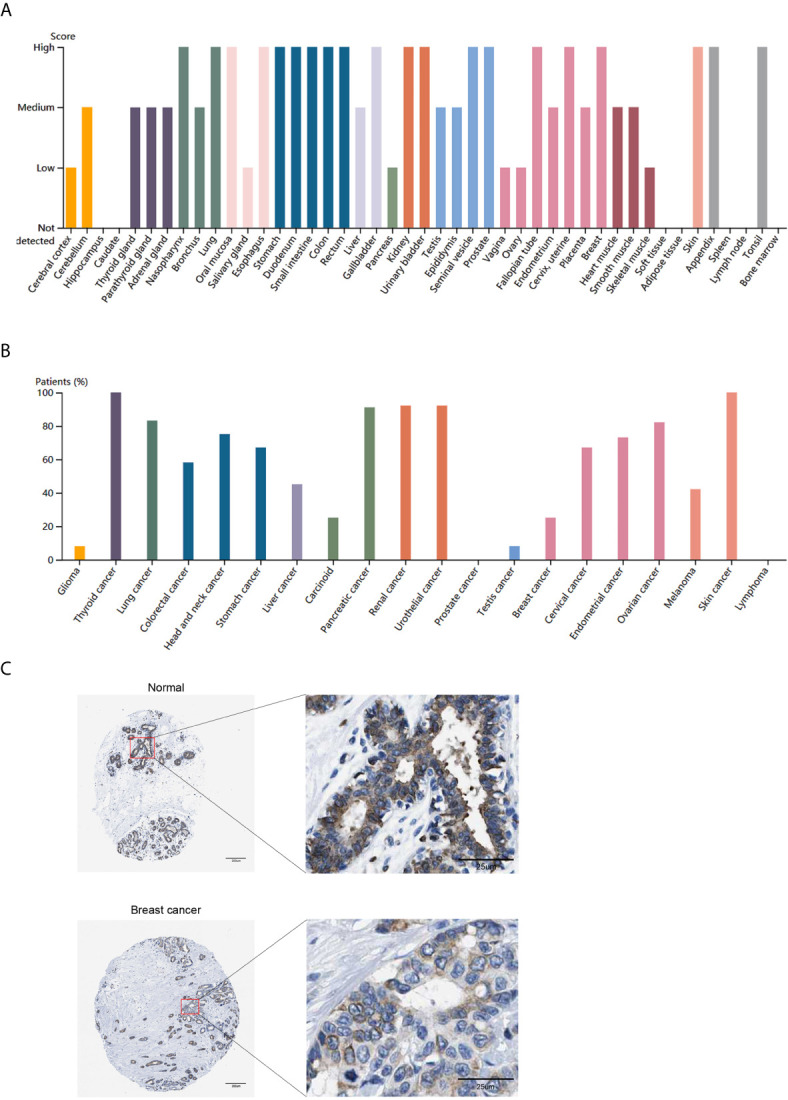
*ITGA3* translation level in different types of tumor tissues and normal tissues. **(A)** The protein level of *ITGA3* in different types of normal tissues in HPA. **(B)** The protein level of *ITGA3* in different types of tumor tissues in HPA. **(C)** Representative IHC images of *ITGA3* in normal breast tissues and breast cancer tissues (The left scale bar, 200 μm; the right scale bar, 25 μm).

Then, we used the HPA database to analyze the protein expression level of *ITGA3*. As shown in **Figure 2A**, the protein level of *ITGA3* was highly expressed in most normal tissues. Except for lymphoma, glioma, testicular cancer and prostate cancer, which were usually weakly stained or negative, most tumor tissues showed moderate to strong membrane and/or cytoplasm positivity, and the final statistics are shown in **Figure 2B**. In addition, the immunohistochemical staining of *ITGA3* in breast tissue and breast cancer tissue is shown in **Figure 2C**.

**Figure 3:**
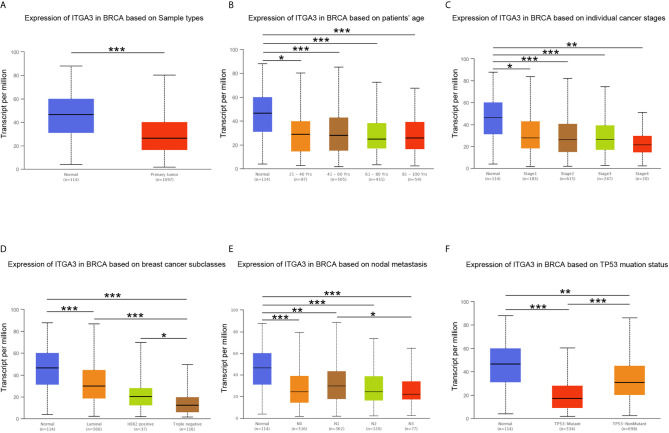
*ITGA3* transcription level in subgroups of patients with BRCA. **(A)** Boxplot of *ITGA3* relative expression based on sample types. **(B)** Boxplot of *ITGA3* relative expression based on patients’ age. **(C)** Boxplot of *ITGA3* relative expression based on cancer stages. **(D)** Boxplot of *ITGA3* relative expression based on breast cancer subclasses. **(E)** Boxplot of *ITGA3* relative expression based on nodal metastasis. **(F)** Boxplot of *ITGA3* relative expression based on TP53 mutation status (The central mark is the median; the edges of the box are the 25th and 75th percentiles. *p < 0.05; **p < 0.01; ***p < 0.001).

In conclusion, the expression of *ITGA3* in breast tissues was higher than that in breast cancer tissues at both the mRNA and protein levels. The mRNA level of *ITGA3* was further analyzed by TCGA-BRCA samples in the UALCAN database. The results showed that based on the analysis of age, tumor stage, breast cancer subtype, lymph node metastasis and *TP53* mutation, the expression of *ITGA3* in breast cancer patients was significantly lower than that in normal controls. As shown in **Figure 3**, the expression of *ITGA3* in triple-negative breast cancer was significantly lower than that in the luminal and *HER2*-positive subtypes. In the lymph node metastasis classification, the expression of N1 was significantly higher than that of N3. In addition, the expression of the *TP53* mutant was significantly lower than that of the nonmutant.

### Prognostic Analysis of *ITGA3* in Cancer Patients

**Figure 4:**
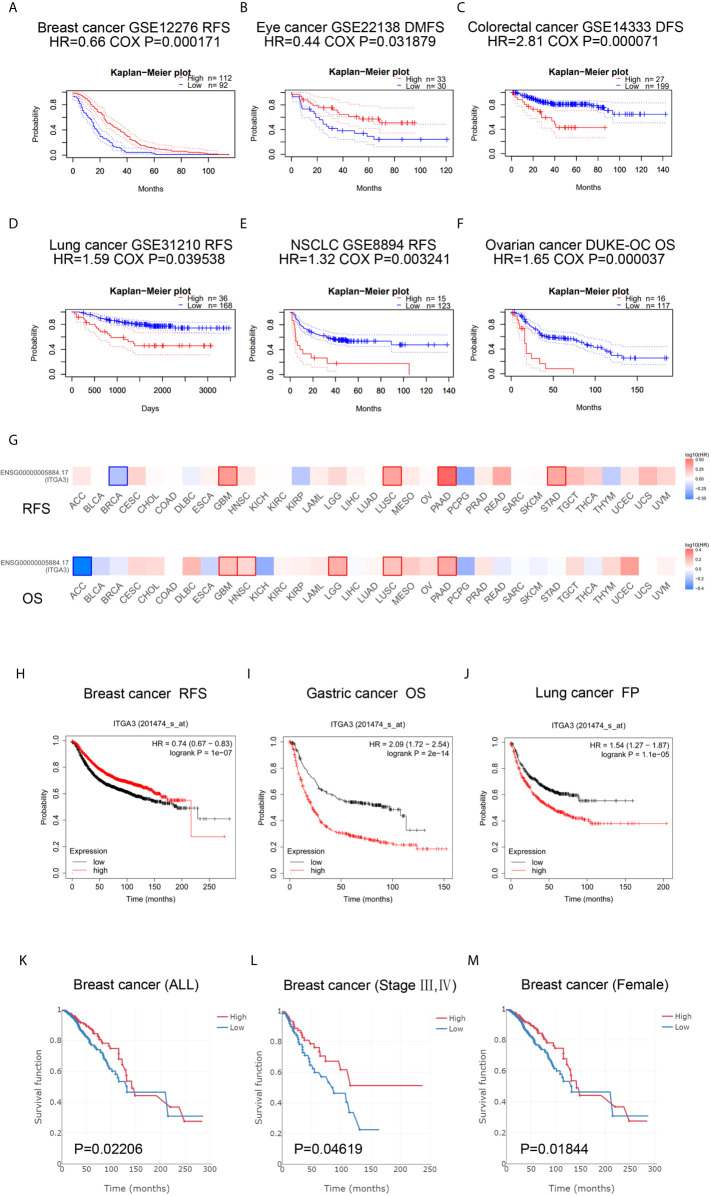
*ITGA3* is associated with survival outcome. **(A–F)** Correlation between *ITGA3* and prognosis of various types of cancer in the PrognoScan. **(G)** Survival map of *ITGA3* in pan-carcinoma. **(H–J)** Kaplan–Meier curves comparing the high and low expression of *ITGA3* in breast cancer (RFS), gastric cancer (OS), lung cancer (FP). **(K–M)** The expression of *ITGA3* was significant for the overall **(K)**, stage III, IV **(L)** and female **(M)** of breast cancer patients’ OS. OS, overall survival; RFS, relapse free survival; DMFS, distant metastasis free survival; FP, first progression. Logrank P-value <0.05 was considered as statistically significant.

We subsequently used the PrognoScan database and GEPIA database to assess the relationship between *ITGA3* expression and cancer patient outcomes. From the PrognoScan database, high expression of *ITGA3* showed favorable prognosis in breast cancer (**Figure 4A**) and eye cancer (**Figure 4B**), and poor prognosis in colorectal cancer (**Figure 4C**), lung adenocarcinoma (**Figure 4D**), non-small-cell lung cancer (**Figure 4E**) and ovarian cancer (**Figure 4F**). In the GEPIA database, as shown in **Figure 4G**, in terms of RFS among 33 types of cancer, *ITGA3* was a high-risk factor in GBM, LUSC, PAAD and STAD, while a protective factor in BRCA (*P <*0.05). For OS, *ITGA3* was a high-risk factor in GBM, HNSC, LGG, LUSC and PAAD, while a protective factor in ACC (*P <*0.05).

Next, the Kaplan–Meier plotter database was used to evaluate the prognosis of *ITGA3* in patients with breast cancer, ovarian cancer, lung cancer and gastric cancer, as well as the prognosis under different pathological subtypes. **Figure 4H** shows that the high *ITGA3* expression group had significantly better RFS in breast cancer (log-rank test, *P <*0.05) than the low expression group. **Figures 4I, J** show that higher expression of *ITGA3* was associated with a poorer prognosis in gastric cancer (OS) and lung cancer (FP). There was no significant difference between the expression of *ITGA3* and the prognosis of ovarian cancer. We used Easysurv to further verify the effect of *ITGA3* on the survival of breast cancer patients’ OS. The results showed that the expression of *ITGA3* was significant for the overall, stage III, IV and female survival functions (**Figures 4K–M**).

These results indicated that in multiple tumor types, the expression of *ITGA3* was significantly associated with a poor prognosis, while in breast cancer, the expression of *ITGA3* was associated with a better prognosis. We further analyzed the prognostic value of *ITGA3* in various subtypes of BRCA (**Table 1**). The results showed that in stages 1, 2, and 3, the expression of *ITGA3* was significantly related to the survival of BRCA, and *ITGA3* served as a protective factor in the prognosis of BRCA. Due to the lack of sufficient samples, its impact in BRCA stage 4 was unclear. Recently, TMB has become an emerging predictive marker for the efficacy of immune checkpoint inhibitors. Tumors with high TMB had a better response to immune checkpoint inhibitors (23). Therefore, we detected *ITGA3* expression in BRCA under different TMB states and found that *ITGA3* could be used as a prognostic biomarker under low TMB conditions.

**Table 1:** Correlation of ITGA3 mRNA expression and clinical prognosis in breast cancer with different subtypes by Kaplan–Meier plotter.

Subtypes		OS		RFS	
		P-value	Hazard ratio	P-value	Hazard ratio
stage	1	0.23	0.55 (0.2–1.48)	**0.017**	0.18 (0.04–0.88)
	2	**0.014**	0.54 (0.33–0.89)	0.081	0.55 (0.27–1.09)
	3	**0.0031**	0.41 (0.23–0.76)	**0.0047**	0.34 (0.15–0.74)
	4	0.66	1.28 (0.43–3.77)	–	–
TMB	High	0.21	0.74 (0.45–1.2)	0.27	0.67 (0.34–1.36)
	Low	**0.0056**	0.49 (0.29–0.82)	**0.044**	0.52 (0.27–0.99)

“–” Lack of enough samples and unsuitable to be analyzed. Bold values indicate P <.05.

### Diagnostic Value of *ITGA3* in BRCA

**Figure 5:**
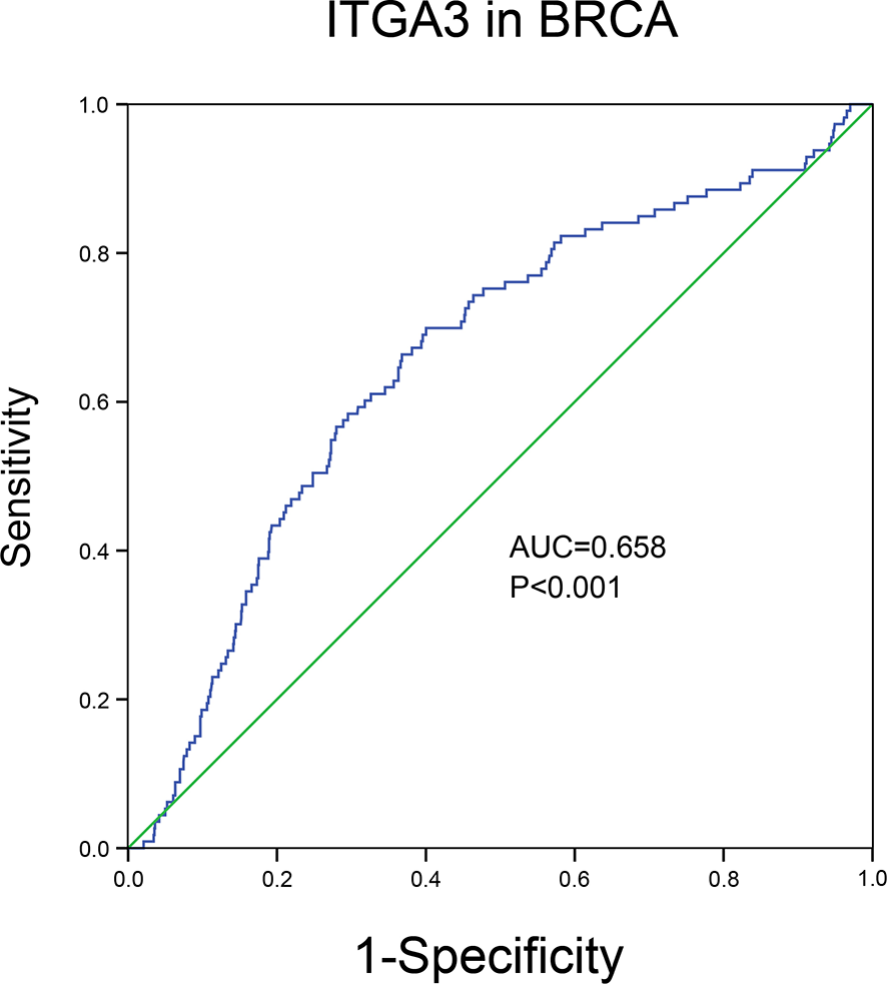
The diagnostic value of *ITGA3* in BRCA based on the TCGA data. P-value <0.05 was considered as statistically significant.

Through the analysis of the expression and prognosis of *ITGA3* in a variety of cancers, we found that *ITGA3* is differentially expressed in several cancers and has a certain impact on prognosis, making it an adverse prognostic factor. Surprisingly, the expression of *ITGA3* in BRCA was lower than that in normal controls and was favorable for prognosis. In view of its prognostic value in BRCA, we used TCGA normal and breast cancer data to generate ROC curves to further analyze the diagnostic value of *ITGA3* in BRCA. **Figure 5** shows that the area under the curve (AUC) area was 0.658, indicating that *ITGA3* has the diagnostic ability to distinguish BRCA from normal controls; subsequently, the diagnostic threshold was further calculated by the Youden index to be 12.505 [transformed by log2(count + 1)]. These results indicated that *ITGA3* was expected to become a diagnostic biomarker for BRCA.

### Genomic Alterations and Methylation of *ITGA3* in BRCA

**Figure 6:**
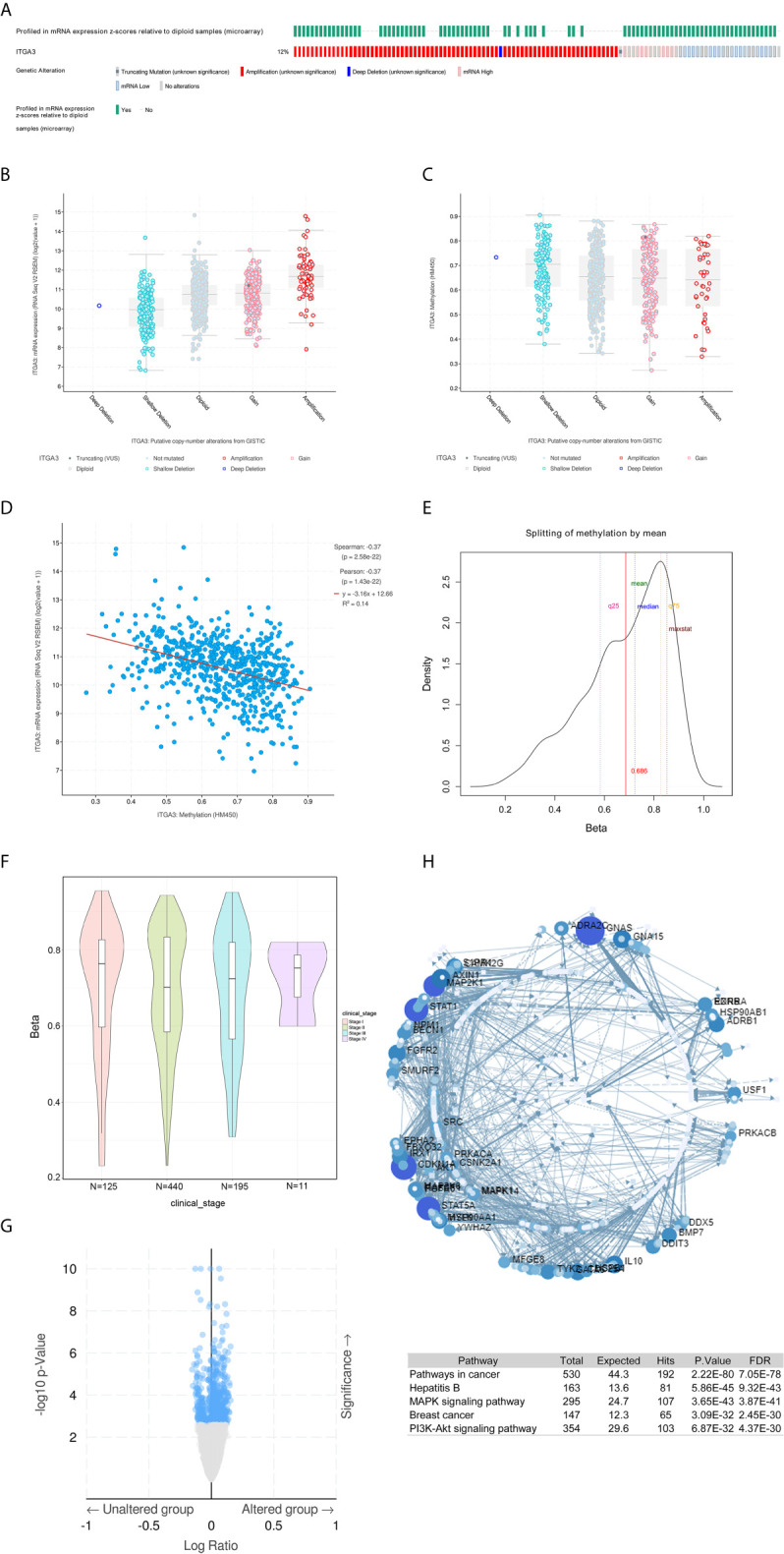
*ITGA3* genomic analysis in BRCA. **(A)** OncoPrint of *ITGA3* alterations in BRCA cohort. The different types of genetic alterations are highlighted in different colors. **(B)** *ITGA3* expression in different *ITGA3* CNV groups. **(C)** *ITGA3* methylation in different *ITGA3* CNV groups. **(D)** The relationship between *ITGA3* promoter methylation level and *ITGA3* mRNA expression in BRCA. **(E)** TSS1500-N_Shore-cg11222053 probe methylation density map. **(F)** TSS1500-N_Shore-cg11222053 probe methylation profiles based on clinical stage. **(G)** Volcano plot of methylated genes between unaltered group and altered group. **(H)** Signaling network of methylated genes with significant differences.

To further explore the mechanism of differential expression of *ITGA3* in breast cancer and normal breast tissue, we used the cBio-Portal tool to analyze the genome of *ITGA3*. We selected the TCGA (Firehose Legacy) of breast invasive carcinoma for analysis, and in **Figure 6A**, *ITGA3* was altered in 113 of 960 (12%) BRCA patients, including mutation in 1 case (0.1%), amplification (AMP) in 62 cases (6.46%), deep deletion in one case (0.1%), mRNA high in 13 cases (1.35), mRNA low in 23 cases (2.4%), and multiple alterations in 13 cases (1.35%). Thus, AMP is the most common type of *ITGA3* copy number variation (CNV) in BRCA. *ITGA3* AMP led to high expression of *ITGA3* (**Figure 6B**). However, *ITGA3* AMP corresponds to a low methylation level (**Figure 6C**), which revealed the potential correlation between the *ITGA3* mRNA expression level and *ITGA3* promoter methylation. Subsequently, we corroborated the correlation, as shown in **Figure 6D**, that the promoter methylation level of *ITGA3* was negatively correlated with the *ITGA3* mRNA expression level. Therefore, we speculated that the low expression of *ITGA3* in BRCA might be due to promoter methylation, which leads to the inhibition of transcription.

Next, we explored the methylation sites of *ITGA3* in BRCA through the Methsurv database. The level of methylation is expressed by *β* value. A *β* value ≥0.6 was considered completely methylated, a *β* value ≤0.2 was considered completely unmethylated, and 0.2–0.6 was considered partially methylated. Finally, it was found that the *β* value of the TSS1500-N_Shore-cg11222053 probe was more than 0.6 (mean) (**Figure 6E**), indicating complete methylation. The probe is located in the promoter region, showing that the promoter of *ITGA3* is methylated. This was consistent with our above speculation: *ITGA3* promoter methylation is one of the mechanisms of *ITGA3* downregulation in BRCA. According to different clinical stages, the median *β* value of stages 1, 2, 3 and 4 was greater than 0.6 (**Figure 6F**).

DNA methylation belongs to the epigenetic category and is an important mechanism for regulating gene expression. As shown in **Figure 6G**, the top three genes with significant differences in methylation between altered group and unaltered group were as follows: *NPM1* (*q*Value = 1.73E−10), *ASNSD1* (*q*Value = 20.1E−09), and *MAK* (*q*Value = 2.72E−08). Next, the genes with significant differences (FDR ≤0.05) were used to construct the signaling network with NetworkAnalyst, as shown in **Figure 6H**. The top five genes with a high degree of difference were *GNAS*, *CDKNA1*, *STAT1*, *STAT5A* and *MAP2K1*. Enrichment with KEGG pathways revealed that the top five highest enrichment pathways were the following: pathway in cancer, hepatitis, *MAPK* signaling pathway, breast cancer and *PI3K-Akt* signaling pathway.

### *ITGA3* Coexpression Gene and Pathway Enrichment in BRCA

**Figure 7:**
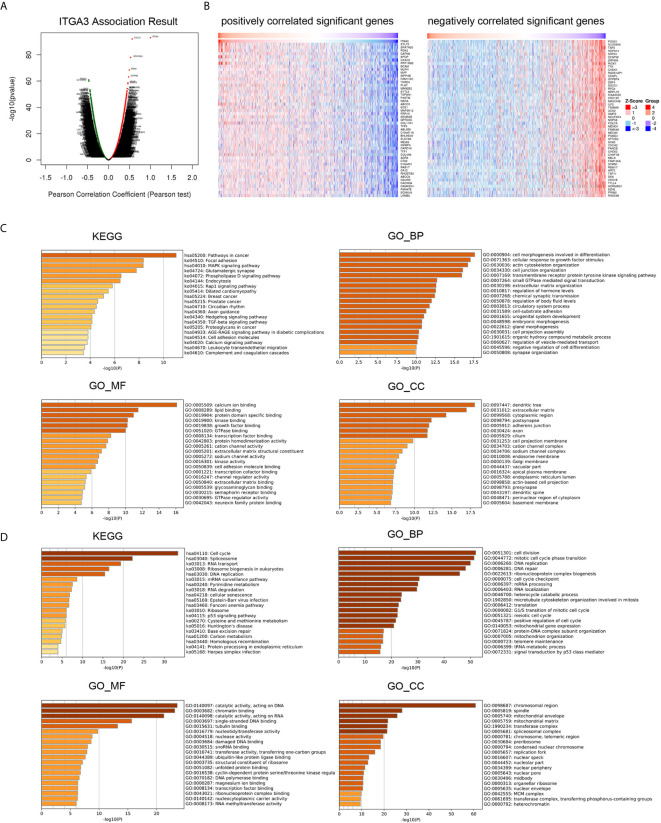
*ITGA3* co-expression genes and pathways enrichment in BRCA. **(A)** Volcano plot of *ITGA3* association result. **(B)** Heat maps showing top 50 genes positively and negatively correlated with *ITGA3* in BRCA. Red indicates positively correlated genes and blue indicates negatively correlated genes. **(C)** KEGG, GO_BP, GO_MF, GO_CC pathway enrichment of positively correlated genes with *ITGA3*. **(D)** KEGG, GO_BP, GO_MF, GO_CC pathway enrichment of negatively correlated genes with *ITGA3*.

To gain insight into the function of *ITGA3*, we next enriched the coexpression gene pathways to visualize the connection between *ITGA3* and coexpression genes. Initially, we used the LinkedOmics database to exhume the *ITGA3* coexpression model in the BRCA cohort. The *ITGA3* association volcano map is shown in **Figure 7A**. *ITGA3* was positively correlated with *XYLT2* (r = 0.564, *P* = 9.85E−93), *SPATA20* (r = 0.526, *P* = 1.11E−78), and *PDK2* (r = 0.497, *P* = 3.65E−69). The heat map of the top 50 genes with significant positive and negative correlations with *ITGA3* is shown in **Figure 7B**.

Then, Metascape was used to analyze the pathway enrichment of *ITGA3* coexpression genes. The pathway enrichment of positively related genes of *ITGA3* is shown in **Figure 7C**, and KEGG pathway analysis showed that positively related genes were involved: pathways in cancer, focal adhesion, *MAPK* signaling pathway, *Rap1* signaling pathway, breast cancer, *TGF-β* signaling pathway, cell adhesion molecules, and leukocyte transendothelial migration. GO_BP (biological process) was mainly related to differentiation, response to growth factor stimulation, cytoskeleton, ECM adhesion and other biological processes. GO_MF (molecular function) was mainly associated with calcium, kinase, growth factor, transcription factor and cell adhesion molecule. GO_CC (cell component) was mainly expressed in dendrites, ECM, adherens junctions and axons. The pathway enrichment of negatively related genes is shown in **Figure 7D**. KEGG showed that the pathways were enriched in cell cycle, spliceosome, RNA transport, DNA replication, and RNA degradation. GO_BP was mainly related to cell division, DNA replication and repair, RNA processing, translation and other biological processes. GO_MF was mainly related to catalyzing the activity of DNA and RNA. GO_CC was mainly expressed in chromosomes and mitochondria.

In conclusion, *ITGA3* coexpressed genes were mainly involved in tumor formation, regulating cell adhesion, migration, proliferation, apoptosis, and immune response.

### Immune Infiltration Analysis of *ITGA3* in BRCA

**Figure 8:**
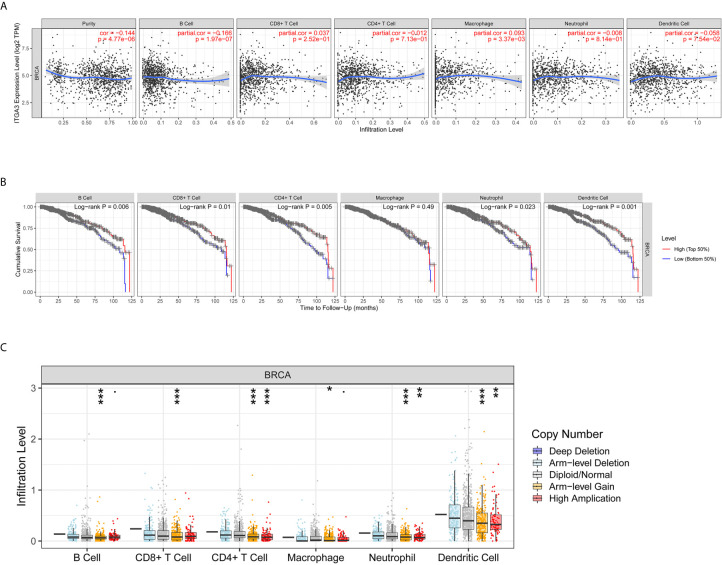
Correlations of *ITGA3* with immune infiltration level in BRCA. **(A)** *ITGA3* expression is significantly related to tumor purity and has significant positive correlations with infiltrating levels of macrophages, and significant negative correlations with infiltrating levels of B cells in LIHC. **(B)** Kaplan–Meier plots of immune infiltration and *ITGA3* expression level in BRCA. **(C)** *ITGA3* CNV affects the infiltrating levels of immune cells in BRCA. (P-value Significant Codes: 0 ≤ *** < 0.001 ≤ ** < 0.01 ≤ * < 0.05 ≤ . < 0.1).

The transformation of breast tissue to breast cancer is usually accompanied by a high level of lymphocyte infiltration. Here, we investigated the relationship between *ITGA3* and TILs in breast cancer. As shown in **Figure 8A**, the expression of *ITGA3* was significantly negatively correlated with tumor purity and B cell infiltration and positively correlated with macrophage infiltration. Next, we further analyzed the effect of immune cell infiltration on the prognosis of BRCA. The results showed that the infiltration of B cells, CD8 T cells, CD4 T cells, neutrophils and dendritic cells was significantly correlated with the prognosis of BRCA (**Figure 8B**). Moreover, the copy number variation of *ITGA3* was significantly correlated with the infiltration levels of six kinds of immune cells (**Figure 8C**), manifesting that the gain or AMP of *ITGA3* was correlated with the infiltration of immune cells and that *ITGA3* may be involved in the recruitment of immune cells.

### Correlation Analysis Between *ITGA3* and Immune Markers

To further explore the relationship between *ITGA3* and immune infiltration, we used TIMER to analyze the correlation between *ITGA3* and multiple immune markers. We selected 16 kinds of common immune cell markers (24, 25) and adjusted the results with tumor purity. As shown in **Table 2**, except for M1 macrophage markers, *ITGA3* was related to most immune markers in BRCA, revealing a significant correlation between *ITGA3* expression and CD8 T cell markers (*CD8A*, *CD8B*), T cell markers (*CD3D*, *CD3E*, *CD2*), Th1 markers (*TBX21*, *IFNG*, *TNF*), Th2 markers (*GATA3*, *STAT6*, *STAT5A*), Tfh markers (*BCL6*, *IL21*), and Th17 markers (*STAT3*, *IL17A*), indicating that *ITGA3* may be involved in regulating T cell responses. In addition, *ITGA3* was negatively correlated with Treg markers (*FOXP3* and *CCR8*) and T cell exhaustion markers (*PDCD1*, *CTLA4*, *LAG3* and *GZMB*), suggesting that *ITGA3* might be involved in immune escape. *ITGA3* was also negatively correlated with B cell markers (*CD19* and *CD79a*), monocyte markers (*CD86*), TAM markers (*CCL2* and *IL10*), and M2 macrophage markers (*CD163* and *MS4A4A*), suggesting that it might be involved in immunosuppression and the regulation of macrophage polarization. In conclusion, these results indicated that *ITGA3* could potentially regulate the recruitment and activation of immune cells in BRCA.

**Table 2:** Correlation analysis between ITGA3 and gene markers of immune cells in BRCA by TIMER.

Description	Gene markers	None		Purity	
		cor	*P*	Partial. cor	Partial. *P*
CD8+ T cell	CD8A	−0.009	0.764	−0.105	**
	CD8B	−0.094	*	−0.192	***
T cell	CD3D	−0.074	.	−0.186	***
	CD3E	−0.048	0.111	−0.160	***
	CD2	−0.079	*	−0.188	***
B cell	CD19	−0.074	.	−0.161	***
	CD79A	−0.036	0.227	−0.140	***
Monocyte	CD86	−0.049	0.105	−0.113	**
	CSF1R	0.121	***	0.058	0.069
TAM	CCL2	−0.034	0.261	−0.080	.
	CD68	−0.012	0.694	−0.061	0.053
	IL10	−0.035	0.246	−0.092	*
M1 Macrophage	NOS2	0.007	0.816	0.003	0.927
	IRF5	0.067	.	0.028	0.381
	PTGS2	0.084	*	0.042	0.181
M2 Macrophage	CD163	−0.024	0.420	−0.078	.
	VSIG4	0.054	0.072	0.000	0.991
	MS4A4A	−0.003	0.919	−0.069	.
Neutrophils	CEACAM8	−0.017	0.583	−0.010	0.745
	ITGAM	0.092	*	0.048	0.134
	CCR7	0.002	0.942	−0.083	*
Natural killer cell	KIR2DL1	−0.047	0.123	−0.110	**
	KIR2DL3	−0.071	.	−0.113	**
	KIR2DL4	−0.140	***	−0.184	***
	KIR3DL1	−0.043	0.158	−0.085	*
	KIR3DL2	−0.078	*	−0.141	***
	KIR3DL3	−0.019	0.524	−0.059	0.061
	KIR2DS4	−0.056	0.062	−0.112	**
Dendritic cell	HLA-DPB1	0.064	.	−0.019	0.552
	HLA-DQB1	−0.032	0.285	−0.102	*
	HLA-DRA	0.009	0.759	−0.070	.
	HLA-DPA1	0.051	0.089	−0.019	0.544
	CD1C	0.130	***	0.061	0.055
	NRP1	0.245	***	0.210	***
	ITGAX	0.024	0.429	−0.031	0.323
Th1	TBX21	−0.063	.	−0.171	***
	STAT4	0.065	.	−0.019	0.544
	STAT1	0.017	0.581	−0.021	0.516
	IFNG	−0.155	***	−0.236	***
	TNF	−0.132	***	−0.155	***
Th2	GATA3	0.353	***	0.401	***
	STAT6	0.373	***	0.362	***
	STAT5A	0.187	***	0.153	***
	IL13	−0.021	0.482	−0.037	0.239
Tfh	BCL6	0.227	***	0.224	***
	IL21	−0.107	**	−0.151	***
Th17	STAT3	0.320	***	0.314	***
	IL17A	−0.045	0.139	−0.083	*
Treg	FOXP3	−0.091	*	−0.154	***
	CCR8	−0.063	.	−0.108	**
	STAT5B	0.314	***	0.302	***
	TGFB1	0.306	***	0.278	***
T cell exhaustion	PDCD1	−0.071	.	−0.165	***
	CTLA4	−0.156	***	−0.242	***
	LAG3	−0.223	***	−0.271	***
	HAVCR2	0.004	0.897	−0.052	0.099
	GZMB	−0.161	***	−0.249	***

Cor, P value of Spearman’s correlation. None, correlation without adjustment. Purity, correlation adjusted by tumor purity. “. <0.05; *<0.01; **<0.001; ***<0.0001” TAM, tumor-correlated macrophage; Tfh, follicular helper T cell; Th, T helper cell; Treg, regulatory T cell.

## Discussion

*ITGA3* is a member of the integrin family, which forms transmembrane integrin with the β1 subunit and is the receptor of fibronectin, laminin, collagen, and epithelial protein. A large number of studies have shown that *ITGA3* can be used as a prognostic indicator for multiple cancers. However, its prognostic effect on breast cancer is still unclear. In this study, we used a variety of databases to explore the expression, survival, prognosis, genomic analysis, coexpression network and immune infiltration of *ITGA3* in BRCA. This study showed that the mRNA and protein levels of *ITGA3* in BRCA were lower than those in normal controls. Further survival analysis showed that low expression of *ITGA3* was significantly associated with poor RFS in breast cancer patients. In addition, tumor pathological stage showed that lower expression of *ITGA3* was associated with poorer OS and RFS in stage 3, indicating that *ITGA3* was related to the prognosis of patients with advanced breast cancer. ROC curves showed that *ITGA3* had significant diagnostic value for breast cancer, indicating that *ITGA3* might be a potential diagnostic biomarker for BRCA. Then, we analyzed the mechanism of *ITGA3* in the prognosis of breast cancer patients. Through pathway enrichment analysis of coexpressed genes, we found that *ITGA3* is related to immune cell infiltration and ECM adhesion. On the one hand, immune infiltration analysis showed that in breast cancer, low expression of *ITGA3* promotes B cell infiltration and M2 polarization. On the other hand, *ITGA3* participates in ECM remodeling. All of these processes can lead to tumor growth and metastasis. Therefore, low expression of *ITGA3* leads to a poor prognosis for breast cancer patients.

By analyzing the expression level of *ITGA3* in breast cancer, we found that the expression of *ITGA3* was lower in breast cancer than in normal controls. Next, we further explored the downregulationed mechanism of *ITGA3* in breast cancer. We proved that there was a significant negative correlation between *ITGA3* mRNA levels and promoter methylation levels through the cBioPortal database. DNA methylation is a kind of chemical modification that can change genetic performance without changing gene sequences and belongs to the epigenetic category (26). DNA methylation is an important mechanism for regulating gene expression, as well as for some genetic diseases and tumorigenesis (27). In mammals, DNA methylation occurs at CpG sites. CpG exists in two forms: one is dispersed in the DNA sequence; the other is found in a highly aggregated state, called a CpG island, which is mainly located in the promoter and 1st exon (28). In normal tissues, most of the scattered CpG is methylated. Except for locations on the inactivated X chromosome, in imprinted genes and in nonexpressing tissue-specific genes, the CpG islands of normal cells are prevented from being methylated (26, 28). The whole genome is divided into four regions: promoter, body, 3UTR and intergenic. The promoter can be subdivided into TSS200, TSS1500, 5UTR and 1st exon (29). Promoter methylation leads to gene silencing and plays a role in carcinogenesis (27, 30). Studies have shown that DNA methylation plays a key role in the development of early gastric cancer, and *ITGA3* methylation is related to mixed gastric cancer (31). We verified the methylation sites of *ITGA3* in BRCA through the MethSurv database, and the results showed that the cg11222053 probe located in TSS1500 had higher methylation. Furthermore, clinical stages 1, 2, 3 and 4 all showed complete methylation (*β* value ≥0.6). Here, we proved for the first time that *ITGA3* exhibits promoter methylation in breast cancer. In summary, *ITGA3* promoter methylation leads to transcriptional silencing, which may be one of the reasons for the downregulation of *ITGA3* in BRCA.

Biological processes often require multiple gene interactions. To further understand the function of *ITGA3*, we constructed pathway enrichment of coexpressed genes, showing that coexpressed genes of *ITGA3* were mainly involved in pathways in cancer, focal adhesion, cell proliferation, apoptosis, differentiation and migration, leukocyte transendothelial migration, and activation of inflammatory reactions and other processes. Previous studies have shown that *ITGA3*, which promotes cells to adhere to the surrounding ECM, initiates the intracellular signaling cascade, and maintains cell survival, proliferation, adhesion and migration (32). *ITGA3* can promote the migration of endothelial cells (33). In addition, *ITGA3* coexpression genes are involved in the pathway of leukocyte transmembrane migration. Studies have shown that α3β1 can mediate neutrophil chemotaxis through the basement membrane (34). Lerman et al. demonstrated that in addition to promoting migration, integrin α3β1 could also mediate the neutrophil inflammatory response in septicemia by cooperating with *TLR2/1* and enhancing the secretion of cytokines downstream of leukocytes (35). High expression of *ITGA3* is associated with lymphocyte invasiveness (36). O’Connell et al. proved that *ITGA3* mediated lymphocyte adhesion and invasiveness (37). These results all indicated that *ITGA3* could promote leukocyte migration and activate inflammation.

The number of TILs is a powerful prognostic factor for breast cancer patients (38, 39). However, the immune system cannot only inhibit the growth of cancer cells but also establish the conditions of the tumor microenvironment to promote tumor growth (40). Different types of immune cells inhibit or promote tumor development. On the one hand, cytotoxic T lymphocytes (CTLs) target tumor cells to exert antitumor immunity. On the other hand, other immune cells are involved in immunosuppression and immune escape. Circulating monocytes are recruited into breast tumors through chemotactic signals and then differentiate into TAMs to promote tumor growth and metastasis (41, 42). Qin et al. demonstrated that B cells suppressed T cell-dependent tumor immunity and the low immunogenicity of tumors was caused by B cells, whose presence in the priming phase results in disabled CD4 T cells that help CTL-mediated tumor immunity (43). We studied the correlation between *ITGA3* and immune cell infiltration in breast cancer by immune infiltration analysis, and the results showed that *ITGA3* was significantly related to B cell and macrophage infiltration. In addition, further correlation between *ITGA3* and immune markers showed that *ITGA3* could regulate the tumor infiltrating immune cell pattern in the tumor microenvironment of breast cancer. Our results showed that *ITGA3* had a negative correlation with B cell markers (*CD19* and *CD79a*), suggesting that the low expression of *ITGA3* in BRCA might promote B cell infiltration and lead to immunosuppression. We further found that *ITGA3* was negatively correlated with monocyte markers (*CD86*), TAM markers (*CCL2* and *IL10*), and M2 macrophage markers (*CD163* and *MS4A4A*), suggesting that *ITGA3* could regulate the polarization of TAMs and promote tumor growth and metastasis. In addition, *ITGA3* was negatively correlated with Treg markers (*Foxp3* and *CCR8*) and T cell exhaustion markers (*PDCD1*, *CTLA4*, *LAG3* and *GZMB*), suggesting that *ITGA3* might be involved in immune escape. These results suggested that *ITGA3* might regulate the infiltration of immune cells in BRCA, which would have a certain effect on the tumor microenvironment.

The tumor microenvironment (TME) includes ECM components, accessory fibroblasts, and proinflammatory cells (44). Extensive remodeling of the ECM during cancer progression causes alterations in density and composition (45). Collectively, the two important modifications within the ECM are stiffness (rigidity) and degradation, and the two processes are related to one another. The ECM is mainly produced by cancer-associated fibroblasts (CAFs). The interaction between CAFs and cancer cells determines ECM stiffness or degradation, which can produce *TGF-β* for ECM stiffness and matrix metalloproteinases (*MMPs*) for ECM degradation; both promote the proliferation, metastasis and angiogenesis of cancer cells. A stiff or rigid ECM is capable of stimulating epithelial cell transformation from normal cells to malignant cancer cells (46). Tumors, by applying physical forces through the stiffened ECM on host tissues, can displace them to enhance cell‐ECM adhesions and disrupt cell–cell junctions, leading to tumor growth and invasiveness (47, 48). In addition, ECM stiffness accelerates tumor progression by blocking the uptake and transportation of drugs into the tumor. ECM stiffness stimulates hypoxic conditions within the TME, which extend the number of leaky vasculatures within tumor microvessels. This leakage structure makes the blood sticky and increases the flow resistance, which makes it difficult for chemotherapy drugs to enter the tumor (49, 50). In addition, an increase in CSC stemness and expansion of the stem cell niche in the TME mediated by ECM stiffness hampers drug penetration into this niche (51). ECM stiffness has been used for initial screening in the diagnosis of breast cancer (45). The integrin content of the subcellular structures acts as a sensor for ECM stiffness, thereby influencing the rate of matrix rigidity (47). Highly stiffened ECM leads to subsequent degradation. Integrin signaling is significant for the formation of invadopodia. Invadopodia release *MMPs* to mediate ECM degradation, which allows cancer cells to obtain invasive characteristics. Integrin receptors promote ECM contents to adhere to invadopodia structures and further penetrate cancer cells from the basement membrane (52). KEGG analysis indicated that *ITGA3* is involved in the *MAPK* signaling pathway, and *ERK1/2* and *JNK*, which are members of the *MAPK* family, can induce cancer cells to generate *MMPs* to degrade the ECM and invade (47).

**Figure 9:**
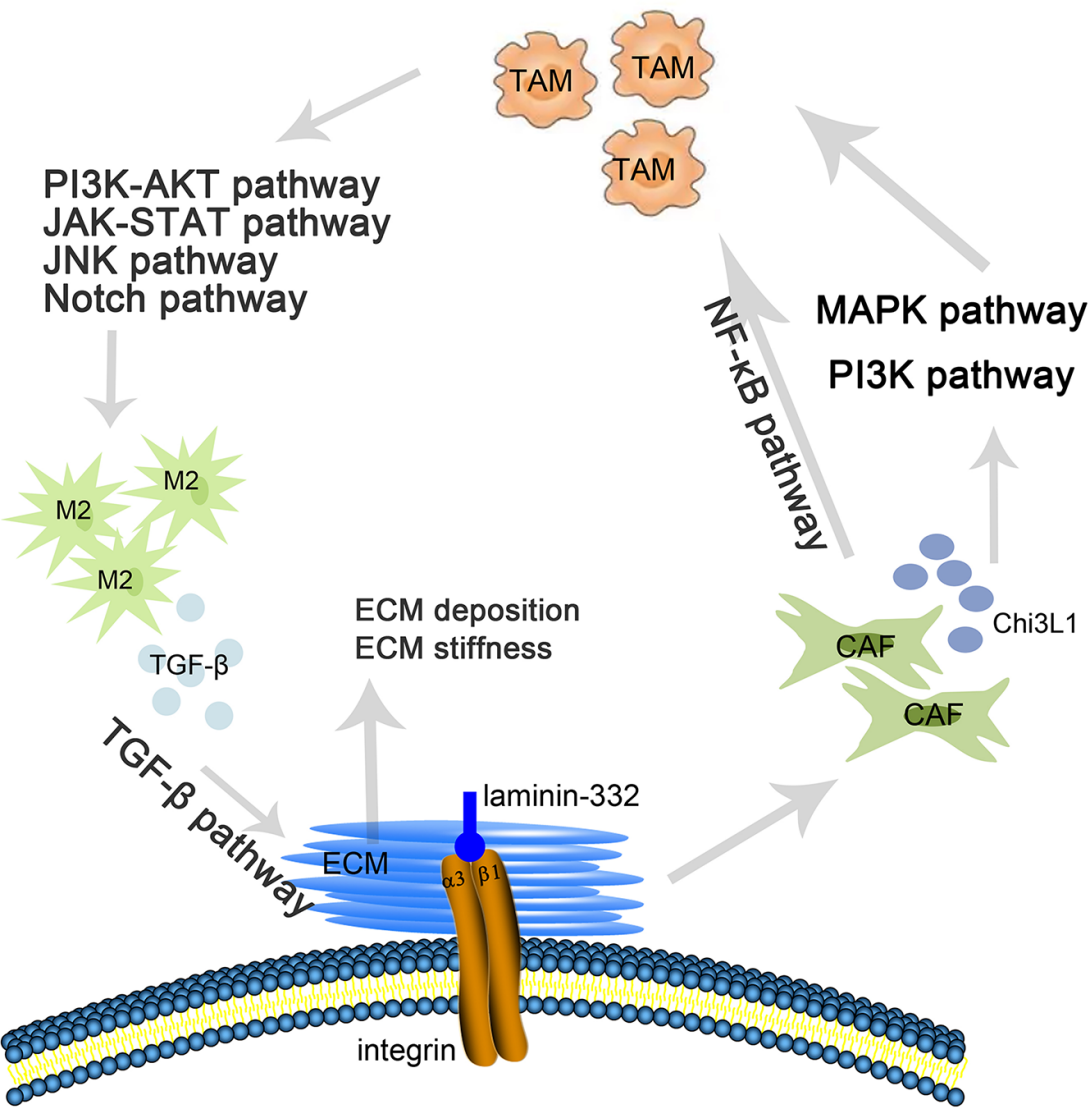
The role of *ITGA3* in regulating ECM and TIL infiltration.

*ITGA3* is as an integrin receptor that can promote the remodeling of the ECM, and changes in the ECM can stimulate integrin signaling to regulate the growth of tumor cells. In addition, *ITGA3* can mediate M2 polarization and promote the stiffness of the ECM. Previous studies have shown that the *α3β1* integrin-laminin-332 interaction of cancer-associated fibroblasts (CAFs) promotes and sustains the differentiation of CAFs and promotes tumor invasion (53). N Cohen et al. showed that CAF-derived *Chi3 L1* mediated *MAPK* and *PI3K* signaling pathways, promoting macrophage recruitment and M2 polarization in breast tumors (54). Additionally, studies have shown that CAFs can recruit macrophages by activating the *NF-κB* signaling pathway (55) and then inducing TAM polarization to M2 macrophages through the *PI3K/Akt* (56), *JAK/STAT* (57), *JNK* (58), and *Notch* pathways (59). M2 macrophages secrete *TGF-β*, stimulating the *TGF-β* signaling pathway in the ECM (60) and inducing ECM deposition, which leads to stiffening of the ECM (61) (**Figure 9**)

In summary, this study provided evidence for the downregulation of *ITGA3* in BRCA, which differed from other types of cancers, due to promoter methylation. In addition, we found that *ITGA3* had prognostic and diagnostic value for BRCA. Exploration of *ITGA3*-related pathways provided important clues for its regulatory mechanism in BRCA. Immunocyte infiltration analysis provides new ideas for ECM and TIL in breast cancer. These results need to be further verified by *in vitro* and *in vivo* experiments. The research in this article is expected to provide a new direction for clinical diagnosis and treatment.

## Data Availability Statement

The original contributions presented in the study are included in the article/supplementary material. Further inquiries can be directed to the corresponding authors.

## Author Contributions

YuL: Conceptualization. YuL and FL: methodology. YuL and XB: software. YuL and YaL: data curation. YuL and CN: resources. YuL: writing—original draft preparation. YuL and DZ: writing—review and editing. XZ and DZ: supervision. XZ: project administration. DZ: funding acquisition. All authors contributed to the article and approved the submitted version.

## Funding

This study was supported by the project of National Nature Science Foundation of China (Nos. 81773076), the project of Nature Science Foundation of Tianjin (No. 19JCYBJC25600) and the basic research cooperation project of Beijing, Tianjin, and Hebei (NO. 20JCZXJC00160).

## Conflict of Interest

The authors declare that the research was conducted in the absence of any commercial or financial relationships that could be construed as a potential conflict of interest.
